# Meningioma microstructure assessed by diffusion MRI: An investigation of the source of mean diffusivity and fractional anisotropy by quantitative histology

**DOI:** 10.1016/j.nicl.2023.103365

**Published:** 2023-03-02

**Authors:** Jan Brabec, Magda Friedjungová, Daniel Vašata, Elisabet Englund, Johan Bengzon, Linda Knutsson, Filip Szczepankiewicz, Danielle van Westen, Pia C. Sundgren, Markus Nilsson

**Affiliations:** a Medical Radiation Physics, Clinical Sciences, Lund University, Lund, Sweden; b F. M. Kirby Research Center for Functional Brain Imaging, Kennedy Krieger Institute, Baltimore, MD, USA; c Russell H. Morgan Department of Radiology and Radiological Science, Johns Hopkins University School of Medicine, Baltimore, MD, USA; d Faculty of Information Technology, Czech Technical University in Prague, Prague, Czech Republic; e Pathology, Clinical Sciences, Lund University, Lund, Sweden; f Neurosurgery, Clinical Sciences, Lund University, Lund, Sweden; g Diagnostic Radiology, Clinical Sciences, Lund University, Lund, Sweden; h Lund University Bioimaging Centre, Lund University, Lund, Sweden; i Department of Medical Imaging and Physiology, Skåne University Hospital, Lund University, Lund, Sweden

**Keywords:** CD, Cell density or cellularity, CNN, Convolutional neural network, DEC, Directionally encoded color maps, dMRI, Diffusion magnetic resonance imaging, DTI, Diffusion tensor imaging, EPI, Echo-planar imaging, FA, Fractional anisotropy, FA_IP_, In-plane fractional anisotropy, H&E, Hematoxylin & eosin, ICVF, Intracellular volume fraction, MD, Mean diffusivity, SA, Structure anisotropy, WHO, World Health Organization, Diffusion tensor imaging, Mean diffusivity, Fractional anisotropy, Cell density, Cellularity, Meningioma

## Abstract

**Background:**

Mean diffusivity (MD) and fractional anisotropy (FA) from diffusion MRI (dMRI) have been associated with cell density and tissue anisotropy across tumors, but it is unknown whether these associations persist at the microscopic level.

**Purpose:**

To quantify the degree to which cell density and anisotropy, as determined from histology, account for the intra-tumor variability of MD and FA in meningioma tumors. Furthermore, to clarify whether other histological features account for additional intra-tumor variability of dMRI parameters.

**Materials and Methods:**

We performed ex-vivo dMRI at 200 μm isotropic resolution and histological imaging of 16 excised meningioma tumor samples. Diffusion tensor imaging (DTI) was used to map MD and FA, as well as the in-plane FA (FA_IP_). Histology images were analyzed in terms of cell nuclei density (CD) and structure anisotropy (SA; obtained from structure tensor analysis) and were used separately in a regression analysis to predict MD and FA_IP_, respectively. A convolutional neural network (CNN) was also trained to predict the dMRI parameters from histology patches. The association between MRI and histology was analyzed in terms of out-of-sample (R^2^_OS_) on the intra-tumor level and within-sample R^2^ across tumors. Regions where the dMRI parameters were poorly predicted from histology were analyzed to identify features apart from CD and SA that could influence MD and FA_IP_, respectively.

**Results:**

Cell density assessed by histology poorly explained intra-tumor variability of MD at the mesoscopic level (200 μm), as median R^2^_OS_ = 0.04 (interquartile range 0.01–0.26). Structure anisotropy explained more of the variation in FA_IP_ (median R^2^_OS_ = 0.31, 0.20–0.42). Samples with low R^2^_OS_ for FA_IP_ exhibited low variations throughout the samples and thus low explainable variability, however, this was not the case for MD. Across tumors, CD and SA were clearly associated with MD (R^2^ = 0.60) and FA_IP_ (R^2^ = 0.81), respectively. In 37% of the samples (6 out of 16), cell density did not explain intra-tumor variability of MD when compared to the degree explained by the CNN. Tumor vascularization, psammoma bodies, microcysts, and tissue cohesivity were associated with bias in MD prediction based solely on CD. Our results support that FA_IP_ is high in the presence of elongated and aligned cell structures, but low otherwise.

**Conclusion:**

Cell density and structure anisotropy account for variability in MD and FA_IP_ across tumors but cell density does not explain MD variations within the tumor, which means that low or high values of MD locally may not always reflect high or low tumor cell density. Features beyond cell density need to be considered when interpreting MD.

## Highlights

• Cell density accounts for MD variability across but not within meningioma tumors.
• Structure anisotropy accounts for in-plane FA variability across and within tumors.
• Vascularization, psammoma bodies, and microcysts influence the MD.
• High and low meningioma tumor cell density can yield similar MD.
• Features beyond cell density need to be considered when interpreting MD.

## Graphical abstract

**Figure:**
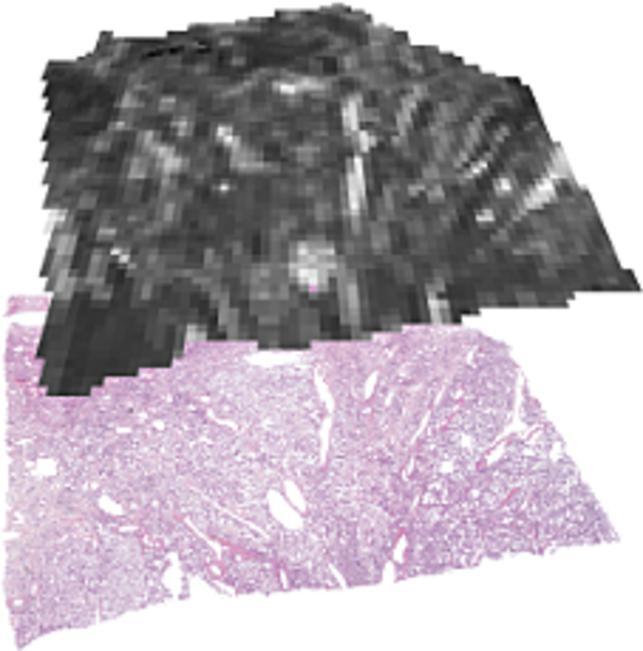

## 1 Introduction

Diffusion MRI (dMRI) is the primary modality for obtaining information on tumor microstructure non-invasively (Stejskal and Tanner, 1965, Brown et al., 2014) and diffusion tensor imaging (DTI) is the most widely applied dMRI method in patients with intracranial tumors. It yields two key parameters: the mean diffusivity (MD) and the fractional anisotropy (FA) (Basser et al., 1994). MD correlates negatively with cell density (CD) in a wide range of tumor types (Sugahara et al., 1999, Gauvain et al., 2001, Chen et al., 2013, Laviolette et al., 2014, Surov et al., 2017). Reduced MD is therefore often interpreted as indicative of viable tumor regions with high CD. Furthermore, the FA reflects the voxel-level diffusion anisotropy and is generally high in white matter due to its highly anisotropic tissue structure. Therefore FA can be used to identify tracts displaced, disrupted, or infiltrated by a tumor (Price et al., 2004, Yen et al., 2009, Jütten et al., 2019).

Although established on the whole-tumor level, it is not clear to which degree the correlation between MD and cell density, or FA and tissue anisotropy, holds quantitatively on a mesoscopic level within individual tumors. There are reasons to believe that both MD and FA can be affected by microstructural features other than cell density and tissue anisotropy. In the case of MD, cellular features such as cell size (Szafer et al., 1995), size of their nucleus (Xu et al., 2009), or membrane permeability (Colvin et al., 2011) have an impact. MD can also be impacted by larger-scale mesoscopic features such as the presence of necrosis (Patterson et al., 2008) or stromal architecture (Squillaci et al., 2004, Yoshikawa et al., 2008). Potentially important features of the stroma include tissue inhomogeneity, the presence of large interstitial spaces, trabecula, nests, and tubular formations, or other complexity of intercellular spaces and junctions. Note that there are examples where MD did not correlate with cell density, such as in renal tumors and breast tumors (Squillaci et al., 2004, Yoshikawa et al., 2008). Furthermore, FA is known to reflect macroscopic (voxel-level) anisotropy, which is lower than the microscopic diffusion anisotropy due to the presence of orientation dispersion (Pierpaoli et al., 1996, Szczepankiewicz et al., 2016). This has been shown to be important in meningiomas, which tend to have high microscopic anisotropy but high orientation dispersion and thus low voxel-level anisotropy (Szczepankiewicz et al., 2016, Nilsson et al., 2020). Thus the interpretation of FA in meningiomas as an indication of tissue anisotropy could be biased by the orientation dispersion of the tumor microstructure (Szczepankiewicz et al., 2015, Brabec et al., 2022). Consequently, it is crucial to understand what affects MD and FA at the mesoscopic level when interpreting local changes in these parameters. However, few studies have investigated the relationship between tumor microstructure as seen by microscopy to what is measured by dMRI on a voxel-to-voxel basis.

Meningiomas are the most prevalent primary intracranial tumor (34% of all intracranial tumors) (Louis et al., 2021). DTI has been proposed for preoperative meningioma classification and consistency estimation, but results have been contradictory (Pistolesi et al., 2002, Gurkanlar et al., 2005, Hsu et al., 2010, Santelli et al., 2010, Lin et al., 2018, Yao et al., 2018). For example, some studies have shown that firm tumors are associated with lower MD values (Yogi et al., 2014, Miyoshi et al., 2020) or with MD values similar to gray matter (Romani et al., 2014). Other studies were not able to reproduce this result (Watanabe et al., 2016) or found that lower MD values are associated with variable consistency (Brabec et al., 2022). Furthermore, higher FA values have been associated with firm consistency (Kashimura et al., 2007, Tropine et al., 2007, Romani et al., 2014), suggesting that firm tumors may contain mainly anisotropic tissue with high microscopic diffusion anisotropy (Kashimura et al., 2007). Other studies, however, did not find such an association (Ortega-Porcayo et al., 2015, Brabec et al., 2022). DTI has also been proposed for the differentiation of atypical, fibroblastic, and other meningioma subtypes (Jolapara et al., 2010, Surov et al., 2015), but both MD and FA have been reported as being similar across a wide range of meningioma types and grades (Brabec et al., 2022). To understand these divergent results, and if possible advice on ways to explain differences between studies, a better understanding of the link between meningioma microstructure and diffusion MRI results is needed.

In this work, we investigated the association between information derived from histology and that obtained from diffusion microimaging of the same specimen across six different types of meningiomas. We examined quantitatively to which degree cell density (CD) and structure anisotropy (SA) from coregistered histology can account for the intra-tumor variability in MD and in-plane FA (FA_IP_), respectively, as observed with dMRI. The FA_IP_ is defined similarly to the FA but it disregards the through-plane anisotropy making comparisons with thin (5 μm) histological slices more straightforward. Similarly to diffusion tensor analysis, the SA reflects the anisotropy in an image and is obtained from structure tensor analysis, which is similar to diffusion tensor analysis, analysis except that the diffusion encodings are replaced by spatial derivatives (Budde and Frank, 2012). To investigate if there were features beyond CD and SA that could explain MD and FA_IP_, we also trained a convolutional neural network (CNN) to predict MD and FA_IP_ from the histology sections. In addition, we qualitatively investigated voxels associated with large prediction errors to identify microstructure features beyond CD and SA that drive large changes in the dMRI parameters.

## 2 Materials and methods

### 2.1 Patients

This study included 16 patients with radiologically diagnosed meningioma tumors scheduled for surgical treatment between 2016 and 2018 at Skåne University Hospital, Lund, Sweden. Inclusion criteria were age above 18 years, histologically confirmed meningioma, and signed informed consent. The study was approved by the Regional Swedish Ethical Review Authority, and all subjects gave their written informed consent to participate in accordance with the Declaration of Helsinki. Table 1 and Table 2 provides a summary of the histopathological evaluation.

**Table 1:** Overview of histopathological classification of meningiomas samples. In total 16 samples were collected. The microstructural assessment was done according to the WHO criteria of 2016 (Louis et al., 2016).

Sample	Type	Grade
1	Transitional	I
2	Chordoid	II
3	Microcystic/Angiomatous	I
4	Meningothelial	II
5	Transitional	I
6	Meningothelial	II
7	Transitional	I
8	Meningothelial	I
9	Fibroblastic	I
10	Clear-cell	II
11	Transitional	I
12	Fibroblastic	I
13	Transitional	I
14	Microcystic/Angiomatous	I
15	Meningothelial	II
16	Transitional	I

**Table 2:** Overview of histopathological classification. In total sixteen samples have been investigated from which eleven were of WHO grade I and five of grade II. Six different meningioma types were included and the most common was a transitional type of grade WHO I. Microstructural assessment was done according to the WHO criteria of 2016 (Louis et al., 2016).

Type	Grade	#
Transitional	I	6
Fibroblastic	I	2
Microcystic/Angiomatous	I	2
Meningothelial	I	1
Meningothelial	II	3
Chordoid	II	1
Clear-cell	II	1

### 2.2 MR imaging and processing

**Fig. 1:**
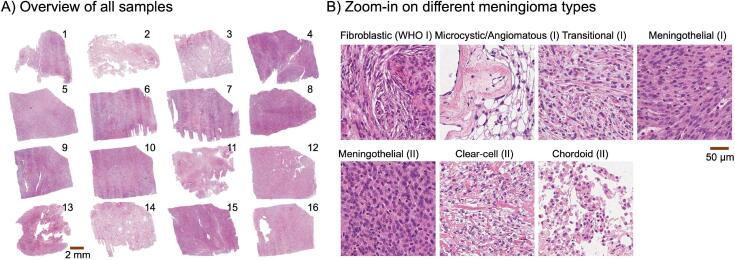
Histology overview. Panel A shows the sixteen meningioma samples that were investigated. Panel B shows zoom-ins on different meningiomas types. Six were transitional, two fibroblastic, two microcystic/angiomatous, three meningothelial (WHO II), one meningothelial (WHO I), one clear-cell, and one chordoid. Microstructural assessment was performed according to the 2016 WHO criteria (Louis et al., 2016).

**Fig. 2:**
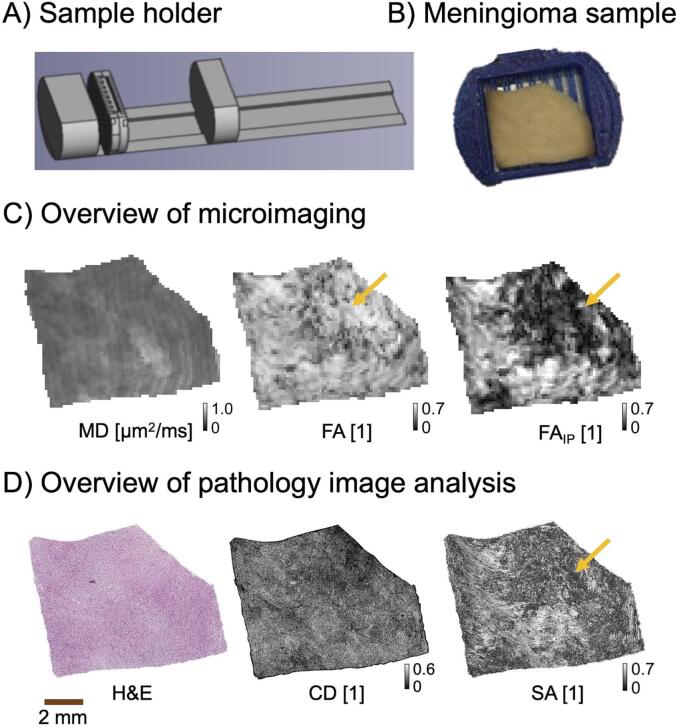
Methods overview. Panel A shows the schematics of the 3D-printed sample holder that was used to facilitate voxel-to-voxel coregistration. Panel B shows a meningioma (sample 5) in the holder. Panel C shows obtained dMRI maps: mean diffusivity (MD), fractional anisotropy (FA), and in-plane fractional anisotropy (FA_IP_). The latter captures only anisotropy within the imaging plane. The upper right part of the tumor has diffusion anisotropy that is dominant in the through-plane direction and therefore FA is high but FA_IP_ low (yellow arrows). Panel D shows a coregistered histology section (H&E stained) that was processed to obtain cell density (CD, cell nuclei count density) and structure anisotropy (SA from structure tensor analysis). (For interpretation of the references to color in this figure legend, the reader is referred to the web version of this article.)

In total 16 tumor samples were obtained after neurosurgical excision and fixated in a formaldehyde solution (4%). The tissue was cut into blocks of approximately 20 × 20 × 2 mm^3^ (Fig. 1A and Fig. 2B) to fit a 3D printed mold (Fig. 2A). Before the MR measurement, the tumor specimens were immersed in a saline solution to allow the water to diffuse throughout the tissue. The specimens were then scanned at a Bruker 9.4T BioSpec Avance III scanner. DTI (Basser et al., 1994) was performed using a 3D-EPI sequence with TR = 1 s, TE = 30 ms, gradient duration *δ* = 4 ms, gradient separation Δ = 15 ms, effective diffusion time t_eff_ = (Δ – δ/3) = 13.6 ms, slices = 185, averages = 10, FOV = 27 × 27 × 37 mm^3^, matrix size = 139 × 139 × 185 voxels, fat suppression on, bandwidth 250 kHz, read offset = 0, phase offset = −2.615, slice offset = 0.101, Coil RF RES 400 1H 059/035 QUAD TR, resolution = 200 × 200 × 200 μm^3^, and with b-values of 100, 1000 and 3000 s/mm^2^ applied in six directions. After the measurement, the tumor samples were again placed in the formaldehyde fixative.

DTI analysis was performed with linear least squares fitting, as implemented in the multidimensional dMRI toolbox (Nilsson et al., 2018b) from which the full-rank diffusion tensor **D_xyz_** was estimated. From this tensor, we calculated FA, MD, and directionally encoded color (DEC) maps. Moreover, the FA_IP_ was calculated from the in-plane components of **D_xyz,_** referred to as **D_xy_**, according to

(1) $$D_{xy}=\left(\begin{array}{cc} D_{xx} & D_{xy}\\ D_{xy} & D_{yy}\; \end{array}\right)$$

Finally, the in-plane FA was computed according to

(2) $$FA_{IP}=\frac{\lambda_{1}-\lambda_{2}}{\lambda_{1}+\lambda_{2}}$$

where λ_1_ and λ_2_ (λ_1_ > λ_2_) are the eigenvalues of the **D_xy_**.

### 2.3 Histopathology

The blocks on which MRI had been performed were embedded in paraffin, sectioned into 5 µm slices, and stained with hematoxylin & eosin (H&E). Each tumor specimen had been diagnosed for tumor type and malignancy grade. This diagnostic procedure adhered to the prevailing WHO criteria of 2016 as part of the clinical routine (Louis et al., 2016) because the data collection took place between the years 2016 and 2018. Sections were then digitalized at a resolution of 0.5 × 0.5 μm^2^. To facilitate coregistration, the sections were consistently taken from one side of the tumor block from the sample holder (Fig. 2AB), which later allowed voxels from MR to be obtained from a similar location.

### 2.4 Coregistration, cell density, and structure anisotropy maps

H&E-stained histology images were coregistered to MR by, first, a rigid coregistration and, second, by a non-linear landmark-based approach. The landmarks were defined on the MD and FA_IP_ maps and then on the corresponding structures in the histology sections. Landmarks were placed at the corners and edges of the sections and also in tumor microscopic features, such as tumor microvasculature, readily discernible in both the histology sections and MR images.

Cell nuclei were segmented from H&E stained images using QuPath (version 0.23) cell detection algorithm (Bankhead et al., 2017). The segmentation was performed using the open-source code available at https://github.com/qupath. Furthermore, the CD was obtained by exporting the cell nuclei centroid positions into a MATLAB environment where the cell nuclei counts were downsampled to match the MR resolution. This was achieved by summing of cell nuclei count over an area corresponding to a single MR voxel and, consequently, the CD map was normalized by dividing by the maximum CD value within the whole sample.

Structure anisotropy was obtained from a structure tensor analysis of the high-resolution histology images using a previously described approach (Budde and Frank, 2012). This consists of computing a structure tensor **H** (Bigun, 1987, Budde and Frank, 2012),

(3) $$H=\left(\begin{array}{cc} H_{\mathrm{xx}} & H_{xy}\\ H_{xy} & H_{yy} \end{array}\right)$$

where *H*_xx_, *H*_yy,_ and *H*_xy_ are partial spatial image derivatives along x or y directions. These were computed as convolutions of the histology image with derivative filters along either the x or y directions and blurred with a Gaussian filter (σ = 0.25 μm). Finally, the obtained structure tensor **H** was smoothed by another Gaussian filter (σ = 15 μm) and downsampled to match the MR resolution (200 µm) which was performed by averaging its eigenvalues within an area corresponding to a single MR voxel. SA was calculated from the eigenvalues λ_1_ and λ_2_ of the downsampled structure tensor **H** as

(4) $$SA=\frac{\lambda_{1}-\lambda_{2}}{\lambda_{1}+\lambda_{2}}$$

where λ_1_ > λ_2_. Finally, the SA maps were smoothed with the same Gaussian kernel as the dMRI maps (σ = 40 μm) to reduce the impact of small coregistration errors. Note that the calculation of SA and FA_IP_ are similar (compare Eq. (1), (2), (3), (4)).

### 2.5 Quantitative assessment of prediction of MD and FA_IP_ from CD and SA across samples

We quantified the degree to which the variability of MD and FA_IP_ can be explicated by CD and SA, respectively, on two different lengthscales. First, we investigated the association on the larger length-scale, that is, for whole tumor samples. We calculated the average SA and CD as well as MD and FA_IP_ within each tumor sample. and calculated R^2^ from the Pearson’s correlation coefficient *r* (R^2^ = *r*^2^) obtained from the analysis between CD and MD as well as between SA and FA_IP_ (*n* = 16 in each case).

### 2.6 Prediction of MD and FA_IP_ from CD and SA within samples

We also investigated the association on the smaller lengthscale, that is, on the intra-tumor level at the 200 µm resolution level. To select only voxels containing tissue only, we drew masks around coregistered MR images as well as the H&E-stained histology sections. The intersect of these two ROIs was then computed and eroded to ensure it contained only the inner parts of the tumor without margins. We will refer to this ROI as the within-tumor ROI (shown in the Supplementary Material for all samples). Scatter plots of MD versus CD and FA_IP_ versus SA from values obtained in the within-tumor ROI showed non-linear relationships (shown in Supplementary Material). Analyses were conducted to identify the function that best explained the relation between them. Three functions were tested for both MD and FA: a first, second, and third-degree polynomial. For MD, yet another function was tested: a second-degree polynomial constrained to be monotonically decreasing with maximal value at minimal CD to mimic the proposed negative association between CD and MD. For FA_IP_, a first-degree polynomial constrained to the origin was tested instead. For comparison, we performed the same procedure also for FA. Results are shown in the Supplementary Material . A second-degree polynomial was best suited in all cases and was used in all subsequent analyses.

**Fig. 3:**
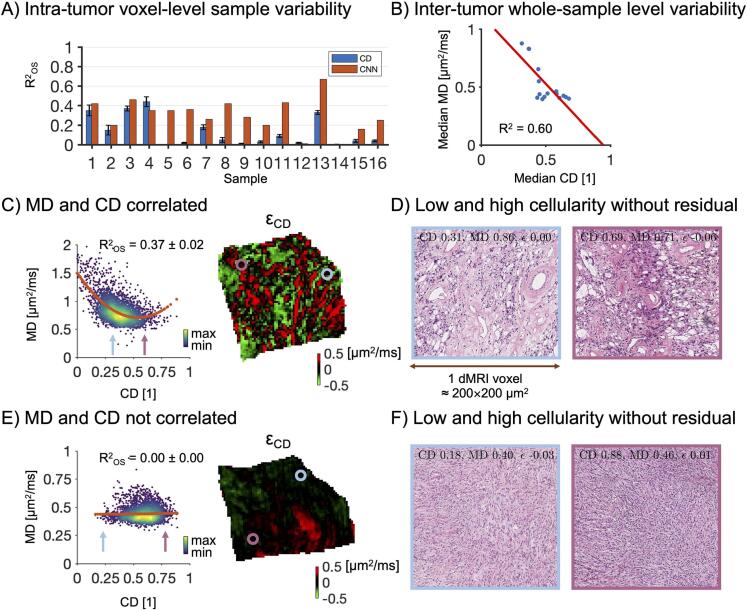
Association between MD and CD. Panel A shows the degree to which the intra-tumor sample variability in MD was explained by a second-order polynomial in CD (R^2^_OS_; blue bars correspond to the median, black error bars show interquartile range) and by the convolutional neural network (CNN; red bars), respectively. Panel B shows the inter-tumor whole-sample average of MD and CD (each blue dot corresponds to a single sample). The variability in MD across tumors is explained well by CD with R^2^ = 0.60. Panel C shows on the left a scatter plot from sample 3 where a strong correlation between MD and CD is present (R^2^_OS_ median ± interquartile range is displayed). High data density is marked by yellow color. A residual map is shown to the right. A voxel of intermediate CD (blue point) and another one with a high CD (purple point) are indicated. Panel D shows their corresponding histology. The voxel with intermediate CD (blue inset) contains tumor stroma with vessels and microcysts, while the one with a high CD (purple inset) has a clearer tumor mass and fewer microcysts and vessels. Panel E shows the same features but for sample 5. Here, the two voxels have similar MD despite having different CDs (low versus high CD as indicated by the arrows in the MD-versus-CD plot). The voxel with low CD appears to have larger cells with larger cytoplasmic volumes than the cells in the voxel with high CD. (For interpretation of the references to color in this figure legend, the reader is referred to the web version of this article.)

### 2.7 Quantitative assessment of prediction of MD and FA_IP_ from CD and SA within samples

To quantify the associations between histology and MRI on the intra-tumor level, we randomly selected 80 % of the voxels as a training set and fitted a second-order polynomial in CD or SA to MD or FA_IP_, respectively, for each modality and each sample. We quantitatively assessed to what degree CD and SA could explain the variability of the MD and FA_IP_ between the measured and predicted MD and FA_IP_ in the remaining 20% of the voxels using the out-of-sample R^2^ (R^2^_OS_) defined as

(5) $$R_{OS}^{2}=1-\frac{{MSE}_{model}\;}{{MSE}_{\mu }\;}$$

where MSE_model_ is the mean squared error (MSE) between the measured and predicted MD or FA_IP_ maps for the case where second-order polynomials in CD or SA were used for prediction, respectively. We also performed the same analysis where we replaced FA_IP_ with FA to test which of the two parameters were best predicted by histology. MSE was calculated as $MSE=\sum {\left(X_{measured}-X_{predicted}\right)}^{2}/n$, in the test set where X is MD or FA_IP_ and *n* is the number of observations. Similarly, MSE_μ_ denotes mean squared errors between the measured and mean of the measured MD or FA_IP_ map in the test set. Note the definition of R^2^_OS_ uses values that were not seen during the fitting, which means that both the nominator and denominator have the same number of degrees of freedom, as opposed to the case where R^2^ is computed for within-sample residuals. The out-of-sample R^2^ is therefore independent of model complexity, which was needed to unify the evaluation of prediction by histology features (CD and SA) and the prediction using a CNN (explained later) as it contains orders of magnitude more parameters. The process was repeated 1000 times with different random selections of the 80/20 split to estimate the uncertainty in R^2^_OS_.

Finally, we also investigated whether a lack of variability in MD or FA_IP_ within the sample could explain a poor association between intra-tumor predicted and measured dMRI maps. We quantified this by calculating the Pearson’s correlation coefficient r between the R^2^_OS_ and the standard deviation of the related dMRI parameter across the sample, respectively (*n* = 16).

### 2.8 Convolutional neural network design

We also investigated the ability to predict MD and FA_IP_ from histology using a convolutional neural network (CNN). The CNN was composed of an EfficientNetV2 network pre-trained on the ImageNet dataset (Tan and Le, 2021) and fine-tuned with additional layers (network architecture overview in Supplementary Material Fig. 5). The network was designed to solve a patch-to-value regression task with the aim to predict either MD or FA_IP_ per voxel using a spatially corresponding patch of 360 × 360 color pixels from the histology images. We used horizontal and vertical image flipping for data augmentation and a train-validation-test split of 60/20/20%, 25 training epochs with early stopping, and a batch size of 32. The number of trainable parameters was 117 787 873. During training, the mean squared error was used as the loss function. Only data from the within-tumor ROI was used in the training to ensure that the model learned tissue features only and not partial volume effects along the edges. The objective of this investigation was to determine whether a CNN could identify histological features beyond CD and SA that have an impact on MD or FA_IP_. Since the purpose was not to learn a general mapping from histology to DTI, the training, and testing were conducted in a generous setting (within each sample rather than across all samples).

### 2.9 Quantitative comparison between image-based feature models and CNN

To compare the performance of the image-feature model utilizing CD and SA and the CNN-based model, we computed two metrics. First, we calculated out-of-sample R^2^_OS_ based on Eq. (5) with MSE_model_ computed using the CNN-based predictions within the test set and compared that to the corresponding number computed using the image-feature models (MSE_model_ = MSE_CD/SA_). Second, we also computed the relative performance of the image feature and CNN models using the relative out-of-sample RR^2^_OS_ according to

(6) $${RR}_{OS}^{2}=1-\frac{{MSE}_{CD/SA}\;}{{MSE}_{CNN}\;}$$

where MSE is calculated for samples in the test set. Note the apparent similarity between R^2^_OS_ Eq. (5) and RR^2^_OS_ Eq. (6). R^2^_OS_ compares the MSE between the predictive model and mean-sample model (MSE_μ_) whereas RR^2^_OS_ compares the image-feature model (MSE_CD/SA_) directly to a benchmark model (MSE_CNN_).

### 2.10 Qualitative analysis by residual maps within samples

To investigate whether additional features apart from CD contribute to the variability of MD, we studied residual maps (the difference between measured and predicted MD). These maps were displayed using a color map where black corresponds to voxels without residual, green to voxels where the prediction was overestimated and additional microstructure features causing lower apparent diffusivity would be needed to counteract the overestimation, and red where it was underestimated and features associated with high apparent diffusivity would be needed to counteract the underestimation.

Similarly, we generated residual maps between MD predicted by the CNN and measured MD, and compared them to those obtained by the CD-based prediction. The purpose was to identify features impacting MD apart from CD. Attention was given to regions where the more general CNN approach had lower residuals than the less flexible CD-based regression approach, as this would indicate that the CNN found histology features to explain an MD deviation whereas the CD-based approach did not.

### 2.11 Data and code accessibility

Analysis code and details of the MRI protocols are available at https://github.com/jan-brabec/microimaging_vs_histology_in_meningeomas. The dMRI data were processed by a software package for diffusion MRI available at https://github.com/markus-nilsson/md-dmri (Nilsson et al., 2018b). Code for cell nuclei detection is available at https://github.com/qupath (Bankhead et al., 2017). Details on the coregistration are available at https://github.com/jan-brabec/microimaging_histology_DIB, ta at hupon request.

## 3 Results

In total, 16 meningioma samples of six different types and two different grades were investigated (Table 1 and Table 2). An overview of sectioned blocks is shown in Fig. 1, together with a display of the microstructural features of the different meningioma types. Examples of dMRI maps of MD, FA, and FA_IP_ coregistered with the high-resolution histology maps are shown in Fig. 2. Note the difference between the conventional FA and FA_IP_ maps (Fig. 2D), where the former captures the overall anisotropy whereas the latter captures the diffusion anisotropy within the imaging plane only. Regions with high FA but low FA_IP_ indicate the presence of elongated cell structures pointing in the direction through the imaging plane. Note that the SA map is highly similar to the FA_IP_ map but not to the FA map.

In the voxel-by-voxel within-sample analysis, CD poorly explained the intra-tumor variability in MD (Fig. 3A, Table 3), with R^2^_OS_ = 0.04 (0.01–0.26); median (interquartile range). The intra-tumor variability was much better explained by the CNN, with R^2^_OS_ = 0.31 (0.20–0.42) and RR^2^_OS_ −0.09 (-0.43–0.04) (overview in the Supplementary Material). In 6 samples, CD-based prediction performed substantially worse than the CNN (RR^2^_OS_ < –0.3 in these samples). In the remaining samples, the CD-based approach explained a similar amount of variability as the CNN. The R^2^_OS_ per tumor correlated with the standard deviation of MD within the sample (r = 0.42, p < 0.05, Pearson’s correlation coefficient), meaning that samples with lower variation in MD showed a tendency towards a weaker association with CD. When averaging the values of both CD and MD across the whole sample and testing for an association across tumors we found a strong linear association with R^2^ = 0.60 (n = 16), although it is noteworthy that 5 out of the 16 samples with low CD and high MD stood out from the rest (Fig. 3B).

**Table 3:** Out-of-sample coefficient of determination (R^2^_OS_) values from the test sets between measured and predicted values. R^2^_OS_ values are in the format median ± interquartile range.

SAMPLE	R^2^_OS_ MD		R^2^_OS_ FA_IP_	
	CD	CNN	SA	CNN
1	0.35 ± 0.06	0.42	0.23 ± 0.03	0.29
2	0.15 ± 0.05	0.20	0.00 ± 0.02	0.00
3	0.37 ± 0.03	0.46	0.10 ± 0.02	0.14
4	0.45 ± 0.06	0.35	0.20 ± 0.02	0.35
5	0.00 ± 0.00	0.35	0.32 ± 0.02	0.34
6	0.01 ± 0.01	0.36	0.22 ± 0.02	0.30
7	0.18 ± 0.03	0.26	0.00 ± 0.00	0.00
8	0.05 ± 0.02	0.42	0.14 ± 0.02	0.18
9	0.01 ± 0.01	0.28	0.30 ± 0.02	0.35
10	0.03 ± 0.01	0.20	0.08 ± 0.01	0.14
11	0.09 ± 0.02	0.43	0.08 ± 0.02	0.27
12	0.02 ± 0.01	0.01	0.02 ± 0.01	0.01
13	0.33 ± 0.02	0.67	0.21 ± 0.02	0.36
14	0.00 ± 0.00	0.00	0.00 ± 0.00	0.00
15	0.04 ± 0.02	0.16	0.20 ± 0.02	0.17
16	0.04 ± 0.01	0.25	0.21 ± 0.02	0.25

To study the relationship between meningioma microstructure and MD, individual samples were investigated. One sample showed a clear negative association between CD and MD within the sample (R^2^_OS_ = 0.36, Fig. 3C). A closer inspection of two voxels with either intermediate or high CD is shown in Fig. 3D. The voxel with intermediate CD and higher MD contains tumor stroma, vessels, and microcysts whereas the one with a high CD and lower MD is characterized by a clearer tumor mass and fewer microcysts and vessels. Another sample showed no discernible association between MD and CD (R^2^_OS_ = 0.00, Fig. 3E). A closer inspection of two voxels with similar MD but either low or high CD from that sample showed that the one with low CD contains cells with a larger cytoplasm volume than the one with a high CD (Fig. 3F).

**Fig. 4:**
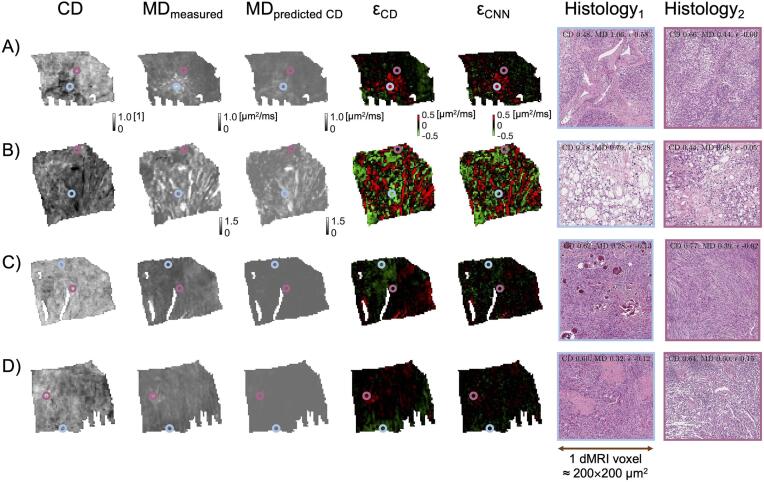
MD is influenced by histological features beyond CD. Columns show maps of the tumor sample and histology zoom-ins of a voxel with a feature associated with an MD poorly predicted by CD (Histology_1_) and a control voxel with an MD well-predicted by CD (Histology_2_). Two residual maps are shown – the first with the residual computed from MD predicted by CD (Ɛ_CD_) and the second with MD predicted by the CNN (Ɛ_CNN_). The color of the border of the zoom-ins matches the color of the circles, which indicate their origin in the sample. Panel A shows that a voxel with underestimated MD (red on the residual map; sample 7) contains tumor vasculature (blue marker), while the control voxel (purple marker) contains no large vessels but only tumor mass. Panel B shows a region with overestimated MD (green color on the residual map; sample 3) that can be linked to tightly packed microcysts (blue). The control voxel (purple) also features microcysts, but fewer. The residual around the microcysts (blue) appears to be dominant when CD is considered for its prediction (Ɛ_CD_) but not for CNN (Ɛ_CNN_). Panel C shows a region with overestimated MD (green on the residual map; sample 9) that could be attributed to psammoma bodies (blue). The control shows no psammoma bodies (purple). Panel D shows that MD can be linked to tissue cohesivity (sample 6). The overestimated voxel (green in the residual map) is associated with tightly-packed tissue with collagen (blue) whereas the underestimated region rather features loose tissue with few vessels (purple). (For interpretation of the references to color in this figure legend, the reader is referred to the web version of this article.)

**Fig. 5:**
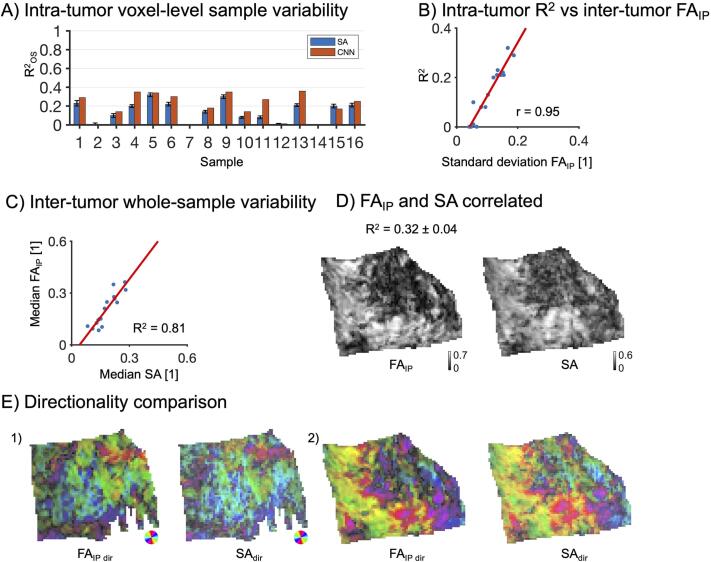
Association between FA and SA. Panel A shows the intra-tumor sample variability in FA_IP_ explained by SA (R^2^_OS_; blue bars correspond to the median; black error bars show interquartile range) and by the convolutional neural network (CNN; red bars). Panel B shows R^2^ from the SA-based regression versus the standard deviation of FA_IP_ values across the whole sample (each blue dot corresponds to a single sample; R^2^ = 0.91). Panel C shows FA_IP_ versus SA averaged across the whole sample and that SA explains FA_IP_ better than the intra-sample analysis (r = 0.95). Panel D shows a visual comparison of SA and FA_IP_ (sample 5). Panel E shows a comparison between the directionality of SA and FA_IP_. Colors indicate directions, while the intensity is modulated by scaling the values by $\sqrt{FA_{IP}}$ and $\sqrt{SA}$. Visually, the predicted and measured anisotropy and directionality of the samples are in strong agreement. (For interpretation of the references to color in this figure legend, the reader is referred to the web version of this article.)

To understand which features could affect MD beyond CD, residual maps were examined. This procedure led to the identification of five types of microstructure features of importance to MD. First, tumor vasculature was associated with an underestimated MD. This is shown in Fig. 4A where an MRI voxel associated with a high residual shows the presence of vessels (histology with a blue border) while a voxel with low residuals lacks them and rather features a solid tumor mass (purple border). Second, tightly packed microcysts were associated with an overestimated MD. This is shown in Fig. 4B, where a voxel containing microcysts (blue border) is compared to a voxel with a denser tumor mass (purple border). This indicates that microcysts act as diffusion restrictions similar to cell bodies. Furthermore, the overestimation from CD was not present when MD is predicted by the CNN (Fig. 4B, residual map Ɛ_CNN_), indicating that the CNN may capture microcysts as a relevant feature. Third, psammoma bodies were associated with an MD overestimated from CD (Fig. 4C). Similar to the case for the microcysts, this bias is absent for the prediction by CNN. Finally, tissue cohesivity may also be relevant for the explanation of the MD. Fig. 4D shows a voxel with tightly-packed tissue with collagen featuring an underestimated MD (blue border) and a voxel with loose tissue and a few vessels with overestimated MD (purple border). This overestimation is more pronounced for the CD-based regression than for the CNN. An overview of residual maps of all samples can be found in the Supplementary Material in Fig. 4 and Fig. 5.

Just as for CD and MD, the SA explained FA_IP_ relatively poorly with R^2^_OS_ = 0.17 (0.05–0.22) (Fig. 5A, Table 3). However, here the per-sample R^2^_OS_ was strongly correlated with the standard deviation of FA_IP_ within samples (r = 0.95, p < 10^–5^, Pearson’s correlation coefficient), meaning that SA did predict FA_IP_ where there was a sufficient feature-driven variation of FA_IP_ within the sample. The CNN displayed slightly higher R^2^_OS_ values than the SA-based regression, with R^2^_OS_ = 0.22 (0.08–0.32) and RR^2^_OS_ = −0.06 (−0.17–0.01) (overview in the Supplementary material), however, unlike in the case for MD prediction by CD, no sample displayed a large discrepancy between the CNN– and SA-based predictions (no sample had RR^2^_OS_ < –0.3). On the whole-tumor level, the association between FA_IP_ and SA was high with R^2^ = 0.81 (Fig. 5C). From a visual perspective, the appearance of SA and FA_IP_ was similar, as illustrated for a sample with a high R^2^_OS_ of 0.32 (Fig. 5D). The directionally encoded maps from dMRI and histology were also similar, as shown in two examples (Fig. 5E). Finally, SA explained almost no variability in FA within tumors, with R^2^_OS_ = 0.03 (0.00–0.07), although it did so across tumors with R^2^ = 0.71. Qualitatively, note that FA_IP_ was closer in visual appearance to SA than FA (Fig. 2C).

**Fig. 6:**
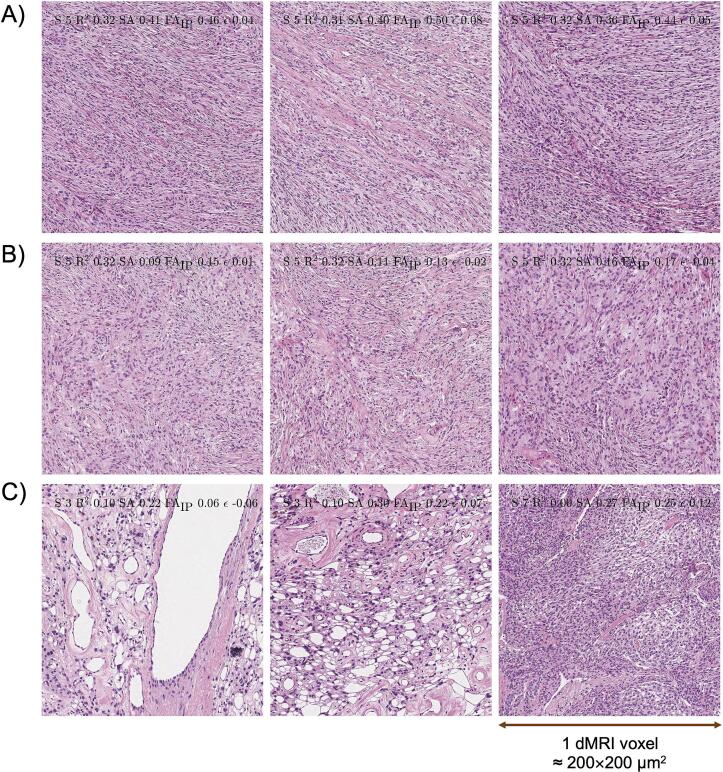
Histology that corresponds to MR voxels with high or low SA and FA_IP_. Panel A illustrates that tissue with elongated structures that are dominantly oriented along one direction yields high diffusion anisotropy (high SA and high FA_IP_). Panel B shows tissue structures oriented without any single preferential direction, which yield low diffusion anisotropy, and thus appear as isotropic tissue (low SA across the whole voxel and low FA_IP_). Panel C shows tissues with boundaries between the tumor and vessels (left), a transition from tumor tissue to microcysts (middle), or tissue looseness with white transparent areas (right). These yield high SA but the structures have little influence on the diffusion and thus yield low to intermediate FA_IP_. This illustrates a limitation of the structure tensor analysis technique.

To analyze the mechanism of the association between FA_IP_ and SA, histology images associated with MRI voxels with high or low FA_IP_ and SA were examined. Voxels with both high SA and high FA_IP_ featured elongated tissue structures oriented more or less along a single direction (Fig. 6A), whereas voxels with both low SA and low FA_IP_ tended to feature high orientation dispersion where the mesoscopic organization appeared more disorganized (Fig. 6B). Furthermore, some voxels featured high SA but low FA_IP_. Such voxels featured boundaries between tumor and vessels, transitions from tumor tissue to microcysts, or loose tissue with white transparent areas (Fig. 6C), which yield high SA due to the strong contrast in the image but are in themselves not likely to have a strong effect on the diffusion. These voxels reflect a limitation in the use of SA as a proxy for tissue anisotropy.

## 4 Discussion

We examined meningiomas ex vivo using both dMRI and histology to understand what microscopic and mesoscopic features of the tumor tissue influence MD and FA from DTI. The analysis was applied to meningiomas of two different grades and six different types (Fig. 1AB), which together displayed the highly heterogeneous microstructure typical for meningiomas (Wiemels et al., 2010). The data allowed us to test the common hypotheses that MD reflects cell density (CD) and that FA reflects tissue anisotropy as quantified by the structure anisotropy (SA). Results were in line with these hypotheses when analyzing data across tumors, however, the hypothesis did not hold within tumors. The cause of the discrepancy was different for MD and FA, however.

Regarding MD, the results indicate that CD alone is insufficient to explain the observed intra-tumor variability in MD. This is exemplified in Fig. 3, where panels C and D show a case where CD is associated with MD whereas panels E and F show a case where it is not. An explanation of the inability of CD to explain the intra-tumor variability in MD could be that it is the intracellular volume fraction (ICVF) unit that determines MD, rather than cell density as defined as the number of cells per volume (example of histology shown in Fig. 3F) (Szafer et al., 1995, Chenevert et al., 2000, Nilsson et al., 2018a, Novikov et al., 2019). To understand this argument, consider that MD is determined by the volume-weighted average of diffusivities in the intra- and extracellular spaces. For cells smaller than approximately 10–15 µm, the intracellular MD is close to zero (Szafer et al., 1995). Therefore, the MD on a voxel level would be given by the product between the extracellular volume fraction (given as 1–ICVF) and the extracellular MD. Voxels with intermediate and high CD could have similar ICVF if their cell sizes were different (e.g. Fig. 3F), and thus similar MD according to this conceptual model. Voxels with very low CD, however, tend to feature loose and necrotic tissue, which leads to lower ICVF and thus higher MD. The comparison across tumors on the whole-sample level supports this idea since on this level the association between CD and MD is driven by the tumors with low to an intermediate CD while for intermediate to high CD there is no discernable association (Fig. 3B).

An alternative explanation to why CD generally failed to explain MD could be that for many samples the measured MD showed little to no variations across the sample, which means that there was no variation to explain (the coefficient of variation of the intra-tumor MD was below 0.2 for 10 out of 16 samples; see in the Supplementary Material). This means that any microscopic feature would yield low R^2^_OS_. However, the CD showed considerable variation in many such cases (the coefficient of variation in CD was below 0.2 for only 4 out of 16 samples; see also Supplementary Material). This means that different CD produced highly similar MD in many samples, which emphasizes the argument raised above that variations in CD do not generally explain variations in MD. Furthermore, the CNN generally explained more variance in MD than the CD (RR^2^_OS_ < 0 in 11 cases), which suggests that features apart from CD contributed to the variation in MD. Examples of such features, identified by inspection of residual maps, include tumor vasculature, psammoma bodies, microcysts, and tissue cohesivity (Fig. 4). We hypothesize that these features are relevant for MD because MD is poorly explained by CD alone and because MD prediction is less biased when the more general and flexible CNN approach is used. This is also in agreement with other studies arguing that features of the mesoscopic stromal architecture influence MD more than CD (Squillaci et al., 2004, Yoshikawa et al., 2008). Furthermore, stromal collagen content (Egnell et al., 2020) or the presence of necrosis may also influence MD (Patterson et al., 2008). Modeling work shows that MD can also be influenced by features of the cells such as their size (Szafer et al., 1995), nuclear size (Xu et al., 2009), or membrane permeability (Colvin et al., 2011). This work hypothesizes that additional microstructural features are of importance (Fig. 4), however, quantifying their effects will be the subject of future work.

The results concerning anisotropy were seemingly similar to those concerning cell density, but subtle differences offer a different interpretation. First, we found quantitively and qualitatively that FA_IP_, which disregards the out-of-plane anisotropy, is a more appropriate measure than FA for comparisons with SA as this metric is calculated from thin (5 μm) histology sections. Similar to MD and CD, FA_IP_ was better explained by SA on the inter-tumor level than on the intra-tumor level (Fig. 5AC). Samples with lower variation in FA_IP_ had markedly lower R^2^_OS_ values (Fig. 5B). This is because the relative importance of noise compared with feature-driven variation is higher for samples with low FA_IP_ than high FA_IP_. Furthermore, samples with a uniform and low FA also showed uniform values and low values of the SA. This stands in contrast to the case for MD and CD, where uniform MD was found even in samples with a non-uniform CD. Furthermore, high SA and high FA_IP_ were associated with the presence of anisotropic tissue structures, and low SA and low FA_IP_ with either isotropic tissue structures or anisotropic tissue structures with high orientation dispersion (Fig. 6AB). This is aligned with prior research (Pierpaoli et al., 1996, Szczepankiewicz et al., 2016), because FA corresponds to the voxel-level average diffusion anisotropy, which is high only in the presence of aligned and elongated microscopic structures and low if either microscopic diffusion anisotropy is low or orientation dispersion is high or both (Szczepankiewicz et al., 2016).

Gaining detailed knowledge of tumor microstructure non-invasively by diffusion MRI is a desirable goal, however, DTI does not attain this goal as our results show that MD and FA are affected by a multitude of different microstructure features and thus lack specific interpretations. To enable the separation of the many features that affect the MD of FA, we need to use diffusion protocols that encode more information than standard DTI protocols (Nilsson et al., 2018a). For example, time-dependent diffusion (Stepišnik, 1993, Chakwizira et al., 2023) could potentially be used to distinguish microcysts from CD because microcysts are circumscribed by an endothelial layer, and their sizes are on average larger than cells. Strong effects of diffusion time could thus indicate the presence of microcysts. Tensor-valued diffusion encoding may also be used to encode for microscopic anisotropy that is independent of tissue orientation dispersion (Szczepankiewicz et al., 2015, Szczepankiewicz et al., 2016, Westin et al., 2016). Improving our ability to map microstructure using such refined dMRI methods may lead to improvements that are relevant to patient care, such as in the preoperative classification or follow-up during therapy of meningioma tumors. For example, improved dMRI methods may help resolve the somewhat divergent results concerning the presurgical prediction of meningioma consistency (Kashimura et al., 2007, Tropine et al., 2007, Romani et al., 2014, Yogi et al., 2014, Ortega-Porcayo et al., 2015, Watanabe et al., 2016, Miyoshi et al., 2020, Brabec et al., 2022).

We identified six potential limitations of the present work. First, the ability to use histology to predict dMRI parameters depends on the accuracy of image coregistration. Herein lies an intrinsic limitation as the MRI voxels were 200 µm thick, whereas the histology sections were only 5 µm thick. The sections were also somewhat deformed during preparation. The influence of the latter limitation was addressed by performing both linear and non-linear registration between the histology images and the dMRI maps. Nonetheless, some of the large residuals seen in highly heterogeneous samples (e.g. Fig. 3C) could be due to a spatial mismatch in the through-slice direction between the two modalities. However, such a mismatch is unlikely to have affected the residual maps in Fig. 4ACD, where highly localized and sample-specific features were clearly related to the residuals. Furthermore, potential registration errors are unlikely to have affected the low R^2^ in samples with uniform MD or FA_IP_, as these simply lacked variance to be explained. Further work is also needed to estimate the variability within the through-plane direction, such as by 2D-stacked histology sections. This could enable a comparison of SA to FA without restricting the analysis to in-plane FA (FA_IP_) only. A second limitation is that features beyond CD that affects MD were identified only qualitatively. Further work is needed to enable the quantification of those features to quantify the strength of their association with MD and this applies also to the proposed and hypothesized relation between intracellular volume and MD. A third limitation is that the meningioma classification was based on Louis et al. (2016) although a newer classification was proposed after the study was closed (Louis et al., 2021). However, a potential reclassification would not affect our conclusions. The only utilization of the classification was to demonstrate the diversity in types and grades and to illustrate the presence of a wide range of microstructural features in the material. A fourth limitation is that the analysis used a second-order polynomial to relate histological image features to measured dMRI parameters. Such a polynomial lacks a biophysical foundation but explained the data reasonably well and served our goal to test for an association between CD and MD or SA and FA_IP_. Future work could use biophysical modeling to better relate histology to MRI. A fifth limitation is that the training a CNN without using a pre-trained part could yield better performance, but prior work reported that fine-tuning of networks pretrained on large sets of images yields better performance for a histology classification task (Vesal et al., 2018). Finally, the results were obtained ex-vivo which may not fully generalize to the in-vivo situation.

## 5 Conclusion

The association in tumors between MD and cell density was present only when comparing across tumors. On the mesoscopic level within tumors, the MD in meningiomas was not determined by the cell density, as several samples demonstrated highly variable cell density but uniform MD. Detailed analysis indicated that MD is also influenced by features such as the presence of large vessels, microcysts, psammoma bodies, and the looseness of the tissue. Furthermore, FA was linked to the tissue structure anisotropy and we found support that it is elevated in the presence of elongated and aligned cell structures in line with previous knowledge.

## CRediT authorship contribution statement

**Jan Brabec:** Conceptualization, Methodology, Formal analysis, Investigation, Visualization, Data curation, Writing – original draft, Writing – review & editing. **Magda Friedjungová:** Software, Formal analysis, Data curation, Conceptualization, Writing – review & editing. **Daniel Vašata:** Software, Formal analysis, Data curation, Conceptualization, Writing – review & editing. **Elisabet Englund:** Resources, Investigation, Project administration, Writing – review & editing. **Johan Bengzon:** Resources, Funding acquisition, Project administration, Writing – review & editing. **Linda Knutsson:** Resources, Funding acquisition, Writing – review & editing. **Filip Szczepankiewicz:** Conceptualization, Methodology, Software, Supervision, Writing – original draft, Writing – review & editing. **Danielle van Westen:** Funding acquisition, Project administration, Writing – review & editing. **Pia C. Sundgren:** Funding acquisition, Project administration, Writing – review & editing. **Markus Nilsson:** Conceptualization, Funding acquisition, Visualization, Methodology, Software, Project administration, Supervision, Writing – review & editing.

## Declaration of Competing Interest

The authors declare that they have no known competing financial interests or personal relationships that could have appeared to influence the work reported in this paper.

## Data Availability

Data will be made available on request.
