# Supplementary material for: Meningioma microstructure assessed by diffusion MRI: An investigation of the source of mean diffusivity and fractional anisotropy by quantitative histology
Source: Neuroimage Clin. 2023 Mar 2;37:103365. doi: 10.1016/j.nicl.2023.103365 (PMC10020119; doi:10.1016/j.nicl.2023.103365)
Supplement: Supplementary data 1 [file mmc1.docx]

# Supplementary material

**
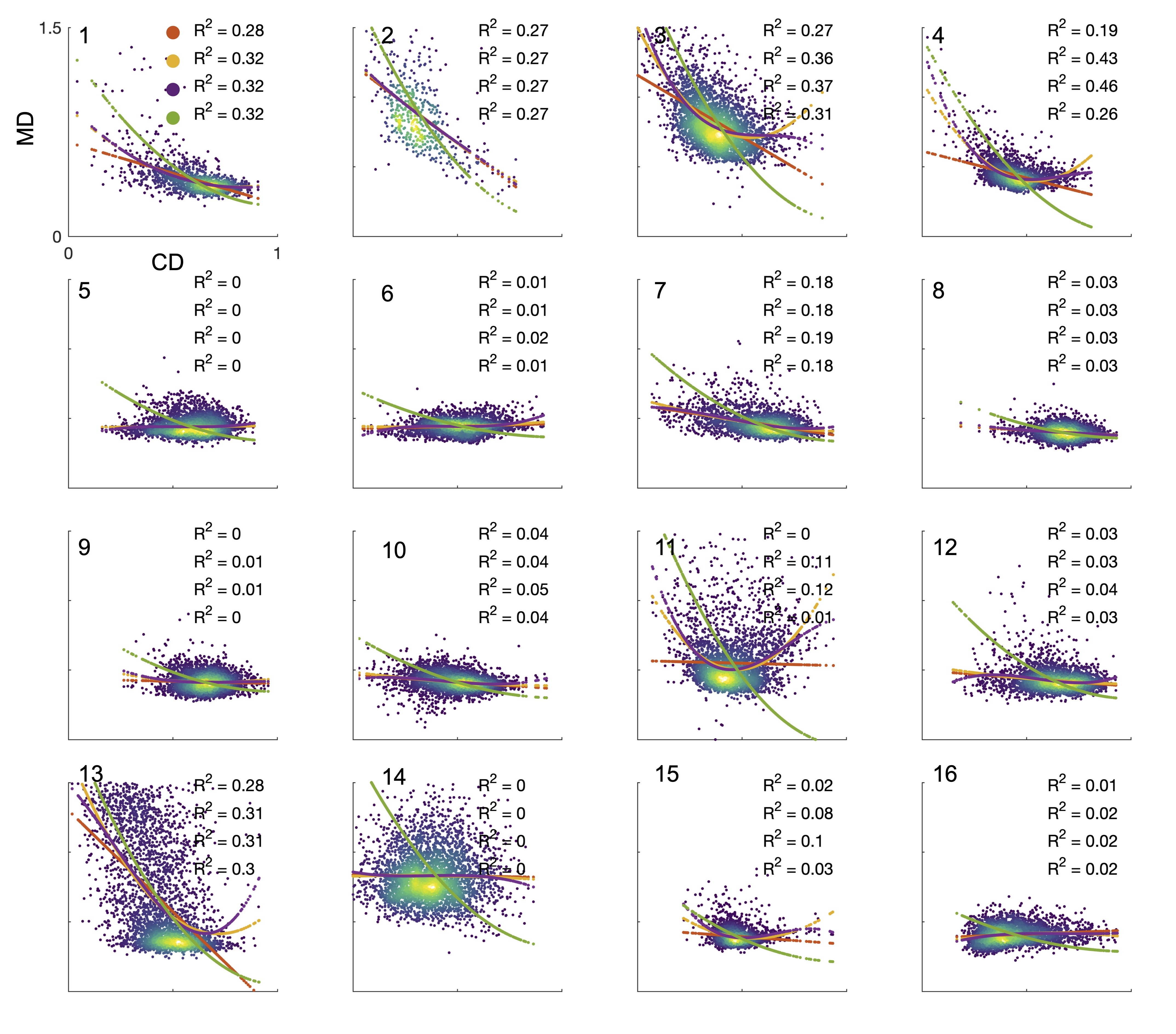
**

**Supplementary Figure 1. Scatter plots and fits of CD vs MD of all samples.** Red color represents a first-degree polynomial, orange color represents a second-degree polynomial and purple color represents a third-degree polynomial. Green color represents a second-degree polynomial constrained to maximal values at minimal CD and monotonically decreasing. Across all samples, we settled upon the second-degree polynomial for further analysis. Sample number in the part of the scatter plot and R^2^ on the right part of the scatter plot (top row linear, second second-degree, third-degree and last row second-degree constrained polynomial).


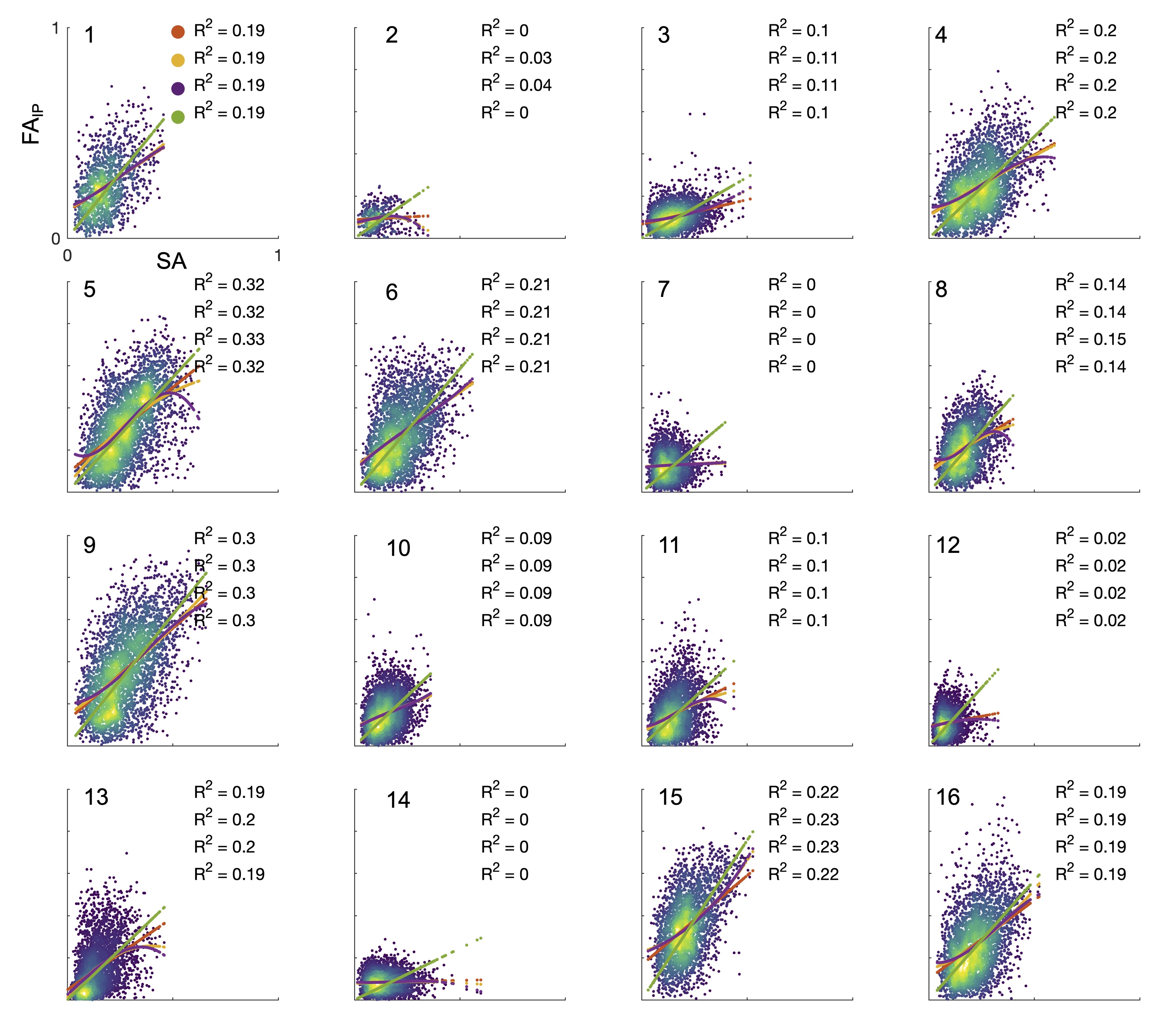


**Supplementary Figure 2. Scatter plots and fits of SA vs FA_IP_ in all samples.** Red color represents a first-degree polynomial, orange represents a second-degree polynomial, purple represents a third-degree polynomial and green represents a second-degree polynomial constrained to the origin (0,0). Across all samples, we settled upon the second-degree polynomial for further analysis. Sample number in the part of the scatter plot and R^2^ on the right part of the scatter plot (top row linear, second second-degree, third-degree and last row first-degree constrained polynomial).


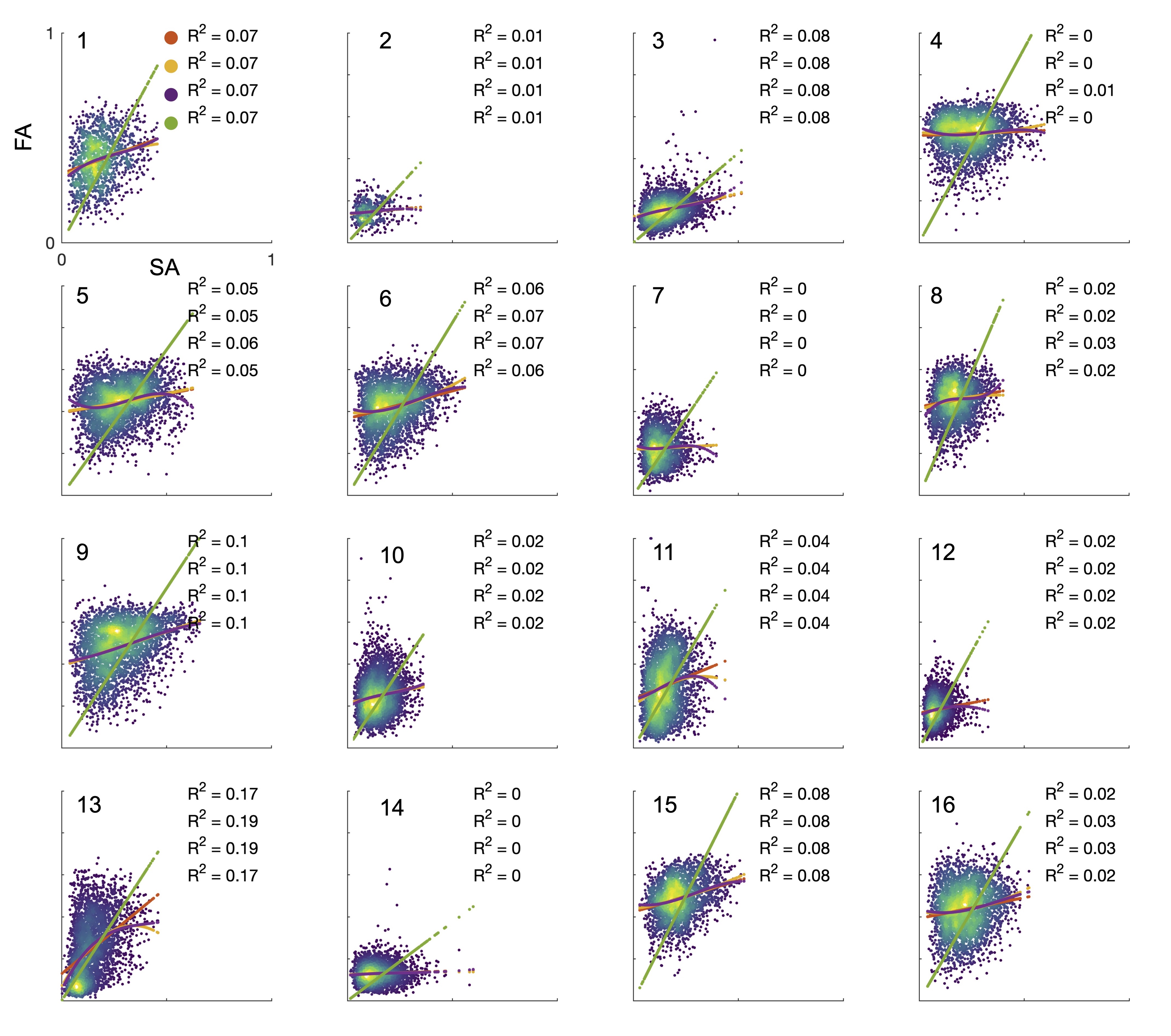


**Supplementary Figure 3. Scatter plots and fits of SA vs FA in all samples.** Red color represents a first-degree polynomial, orange represents a second-degree polynomial, purple represents a third-degree polynomial and green represents a second-degree polynomial constrained to the origin (0,0). Across all samples, we settled upon the second-degree polynomial for further analysis. Sample number in the part of the scatter plot and R^2^ on the right part of the scatter plot (top row linear, second second-degree, third-degree and last row first-degree constrained polynomial).


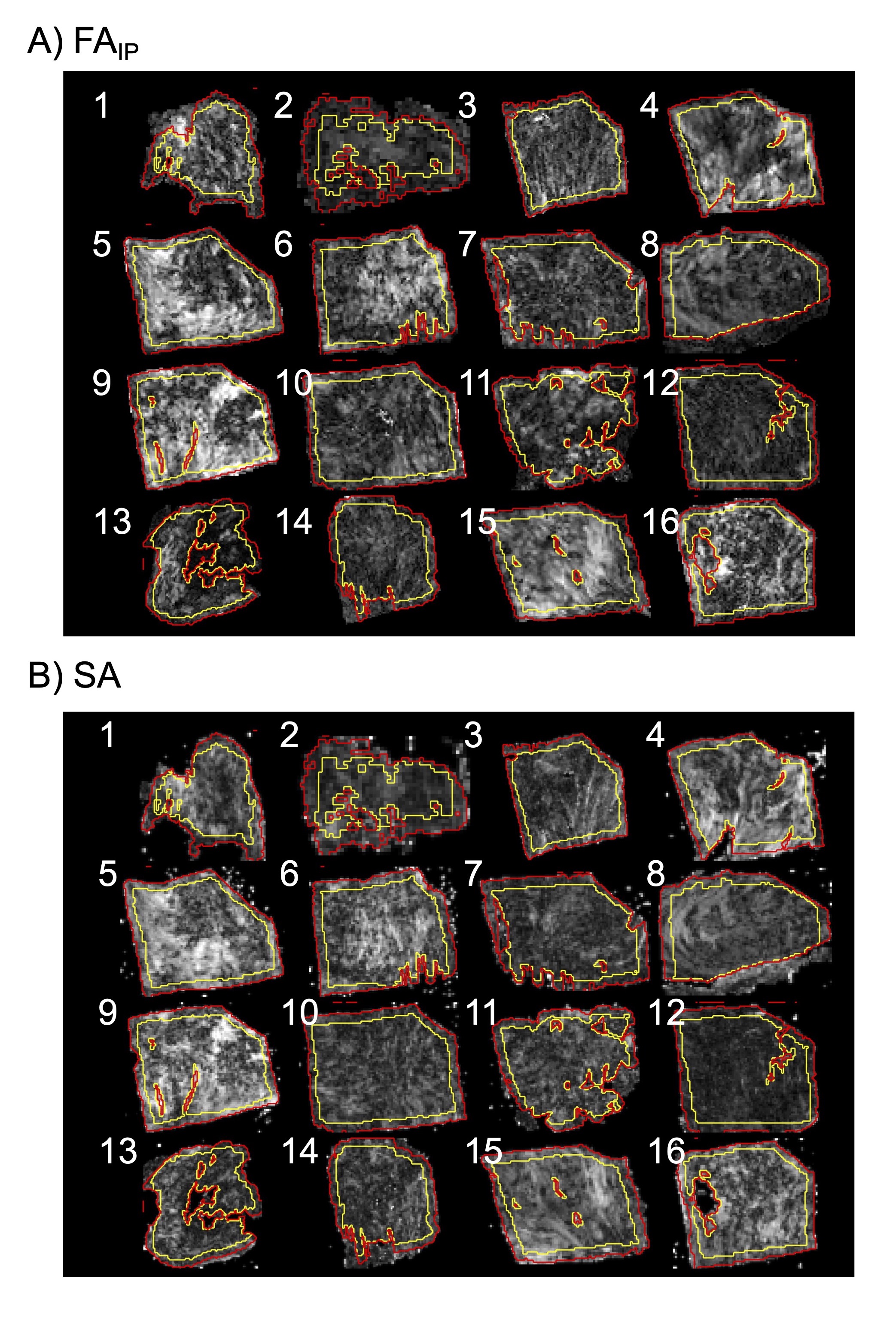


**Supplementary Figure 4. Definition of region-of-interests (ROIs).** Masks around coregistered MR images and around H&E-stained histology was drawn. The mask around histology was downsampled to the MR resolution and a ROI around the whole image was created (red ROI). This was further eroded to contain only parts inside of the tumor excluding the tumor margins (yellow ROI). This ROI (yellow) was chosen for all analysis both quantitative and qualitative.


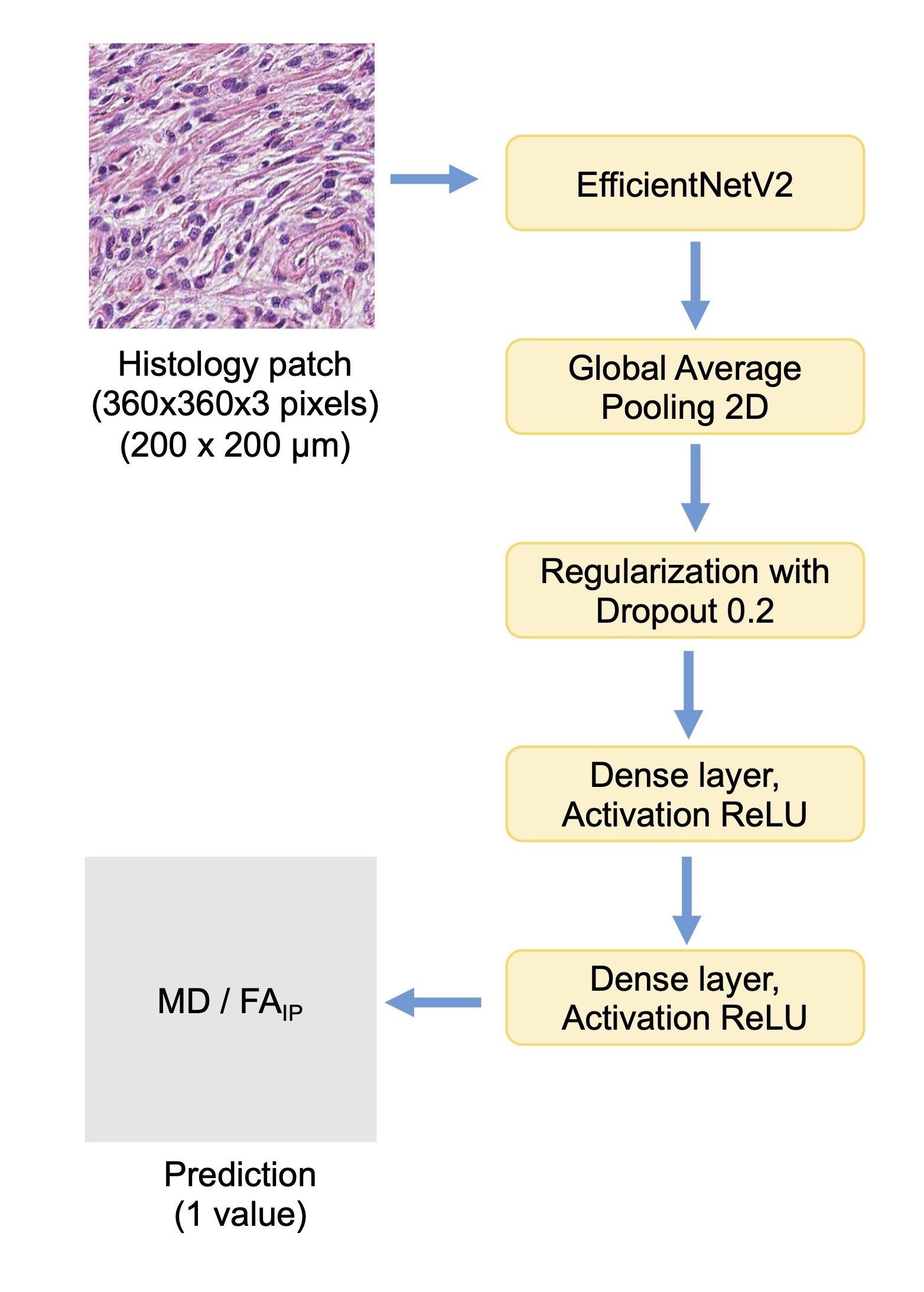


**Supplementary Figure 5. An overview of the convolutional neural network (CNN) architecture.**


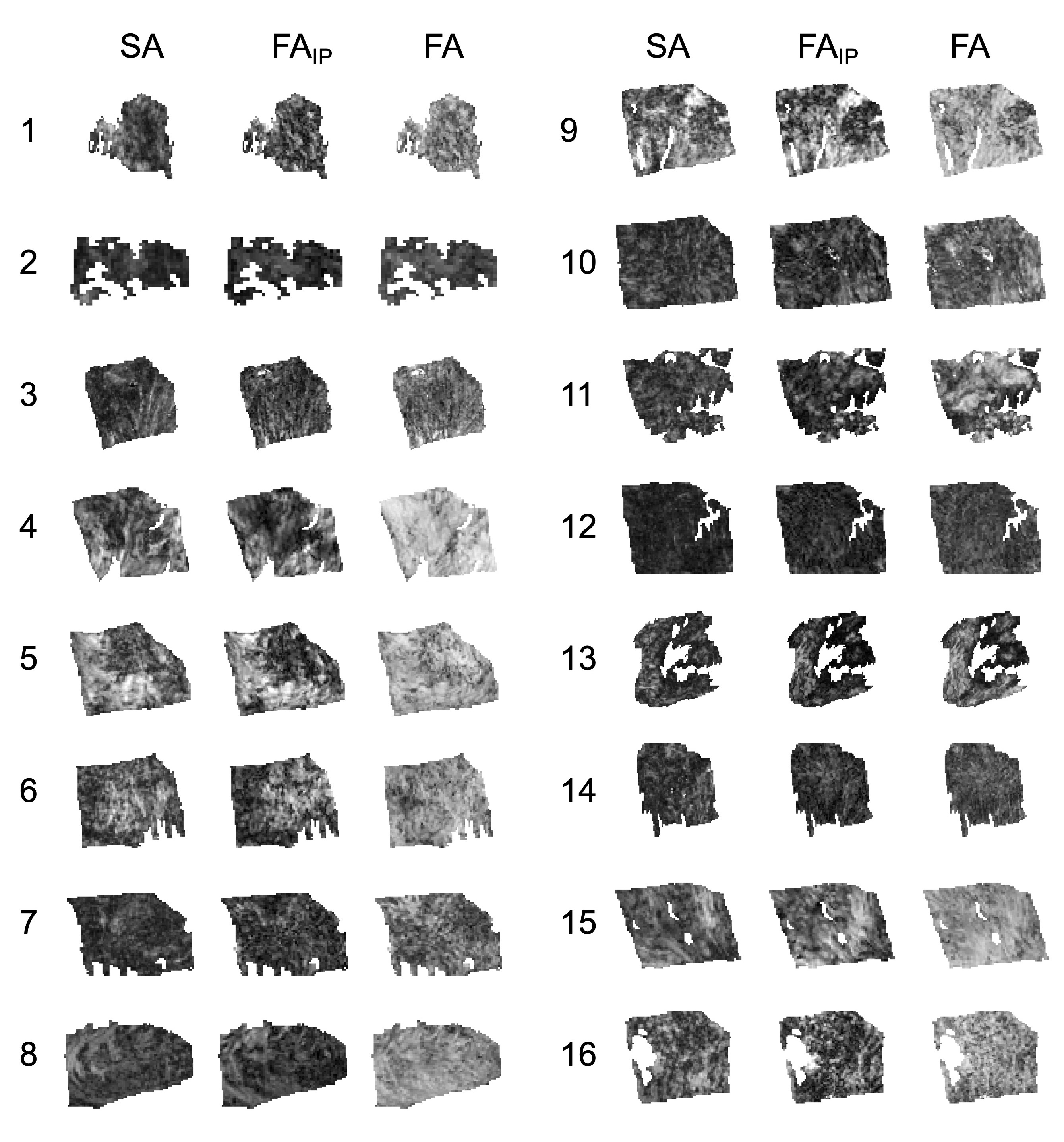


**Supplementary Figure 6. Qualitative comparison between SA, FA and FA_IP_.** The figure shows an overview of all samples with SA with measured FA and FA_IP_. Qualitatively, FA_IP_ is closer to the FA. The FA_IP_ maps are scaled across samples to have similar intensity. The SA maps are scaled to have similar intensity when compared to the FA_IP_ maps for each sample. The FA maps have the same scaling as FA_IP_ maps.


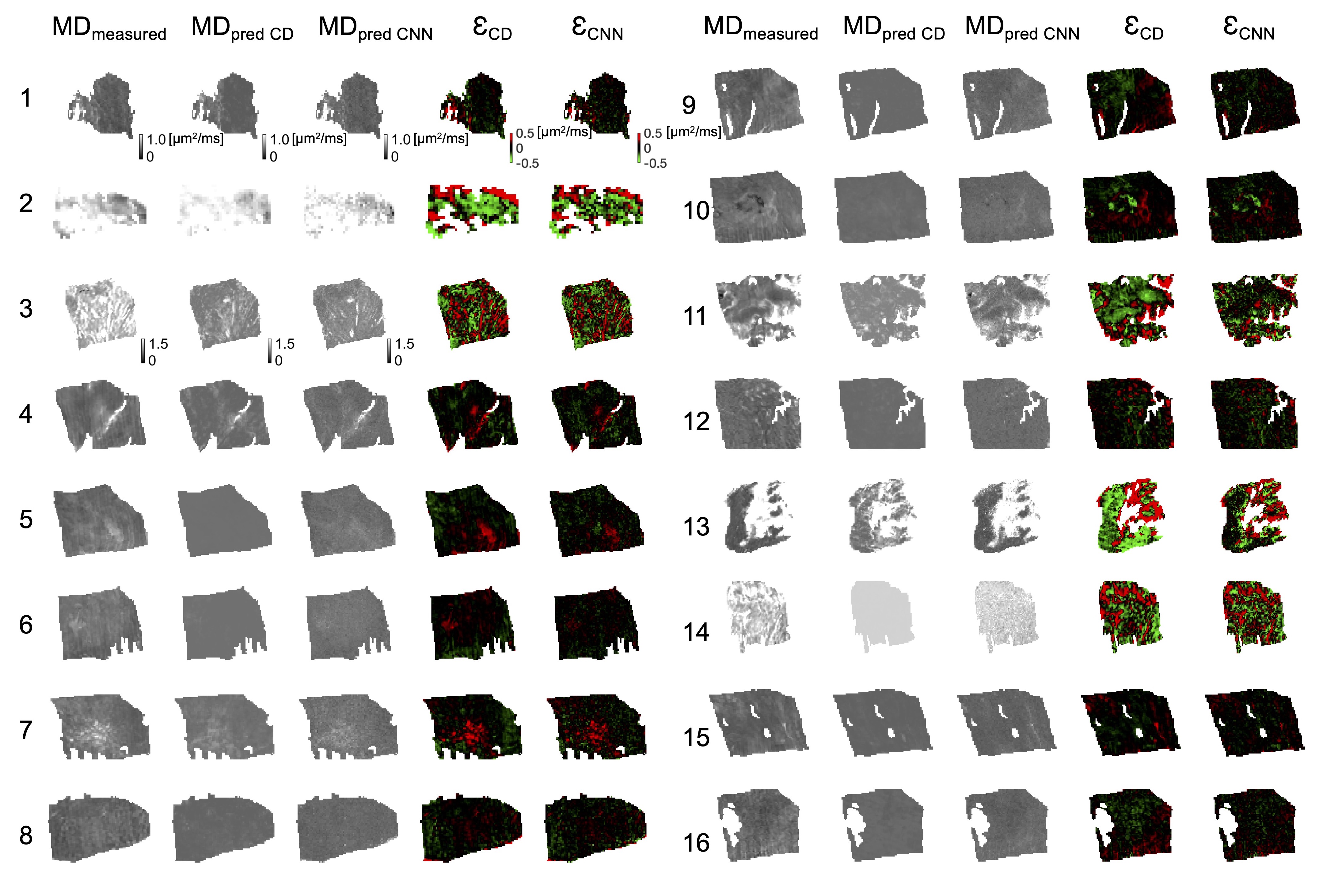


**Supplementary Figure 7. Predicted MD by cell density (CD) and convolutional neural network (CNN) together with residual maps.** The figure shows an overview of all samples with measured MD and MD predicted by CD (MD_pred CD_) or CNN (MD_pred CNN_) as well as their residual maps (measured – predicted MD) of CD (Ɛ_CD_) and CNN (Ɛ_CNN_) predictions.


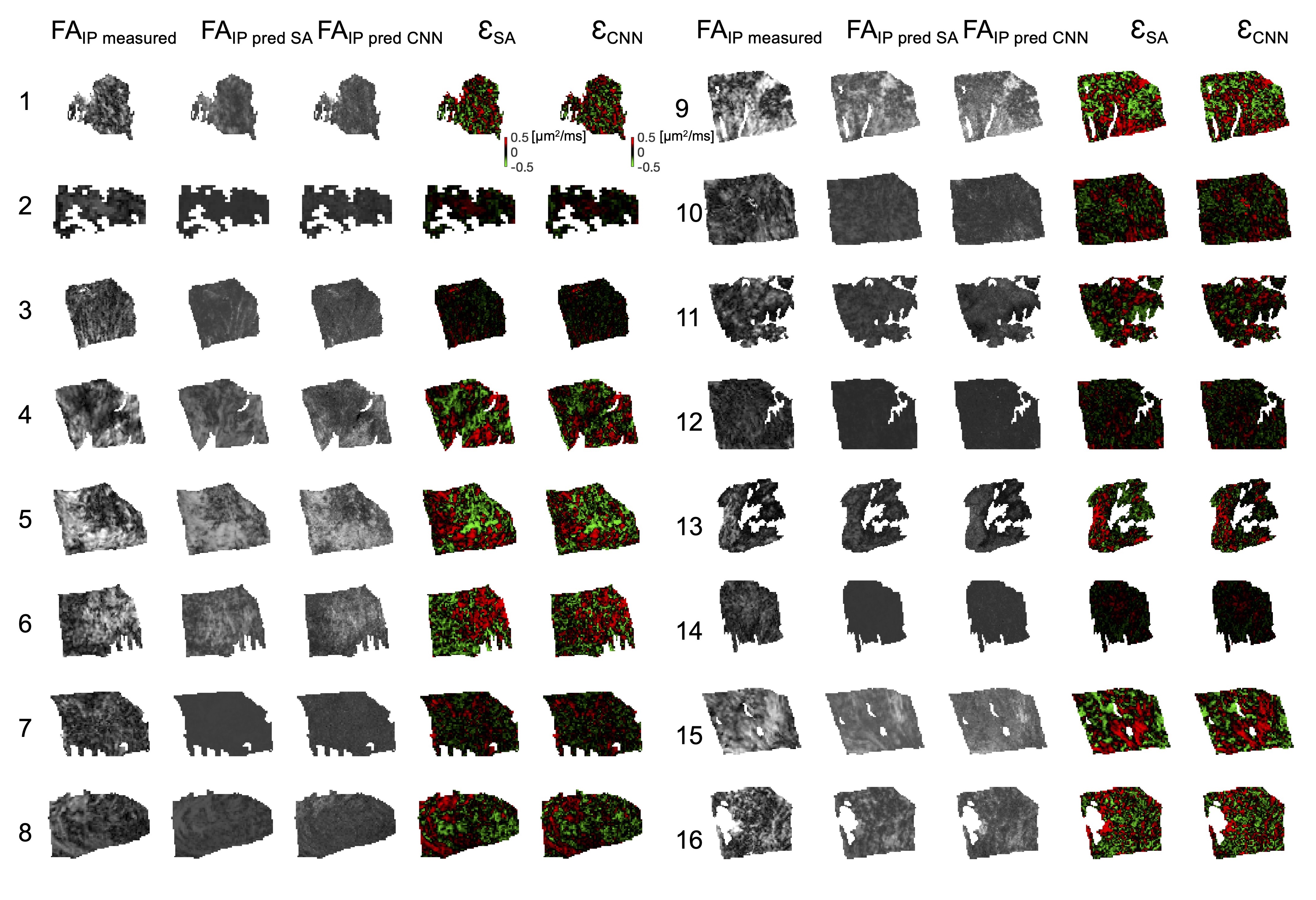


**Supplementary Figure 8. Predicted FA_IP_ by structure anisotropy (SA) and convolutional neural network (CNN) together with residual maps.** The figure shows an overview of all samples with measured FA_IP_ and FA_IP_ predicted by CD (FA_IP_ pred CD) or CNN (FA_IP_ pred CNN) as well as their residual maps (measured – predicted FA_IP_) of SA (Ɛ_SA_) and CNN (Ɛ_CNN_) predictions. The FA_IP_ maps are scaled across samples to have similar intensity but for each sample, the FA_IP_ maps (measured as well as both predicted maps) have the same scaling.


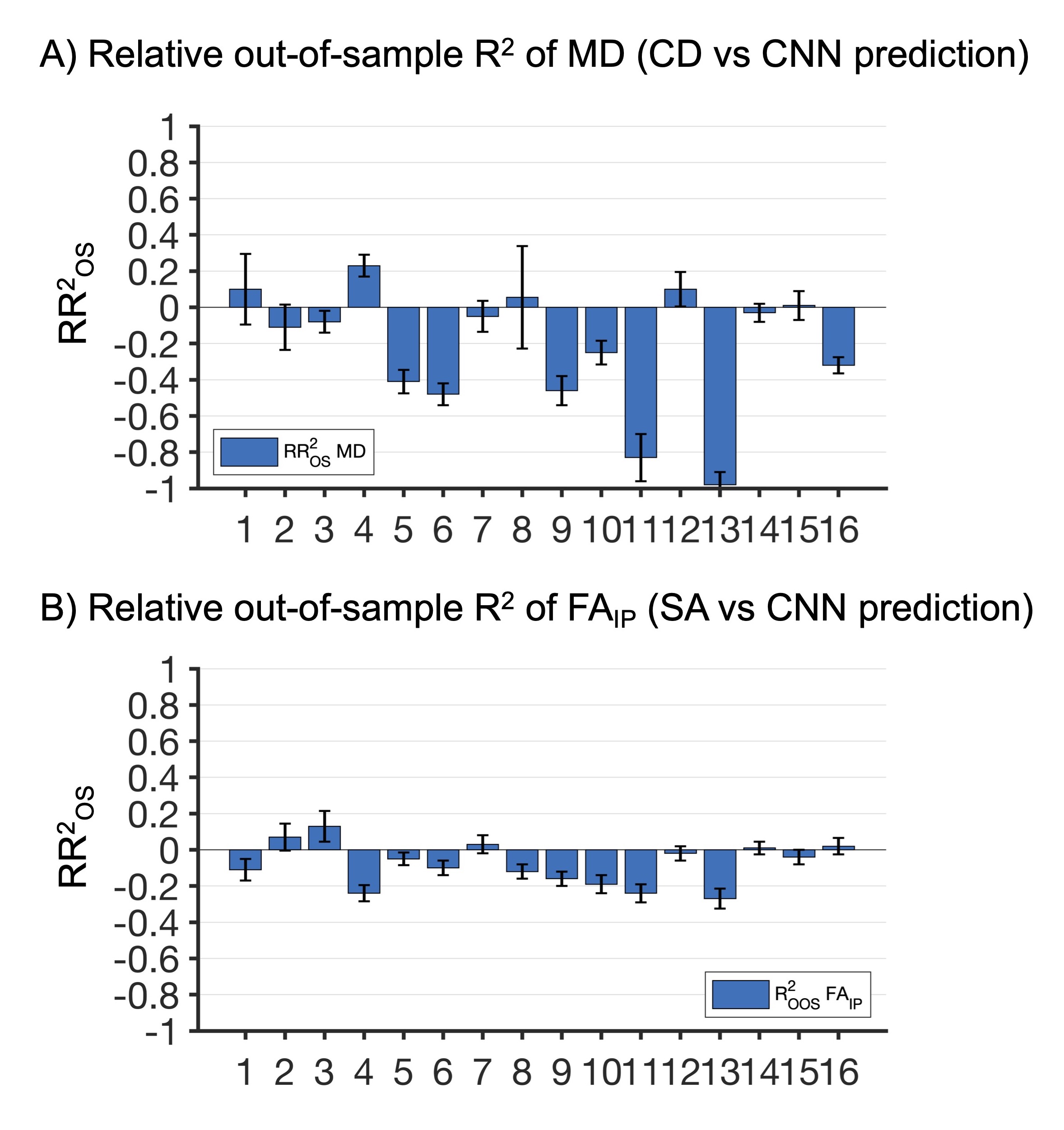


**Supplementary Figure 9. Relative out-of-sample R^2^ (RR^2^_OS_)of MD (panel A) and FA_IP_ (panel B).** RR^2^_OS_ was calculated according to the Eq. 6.


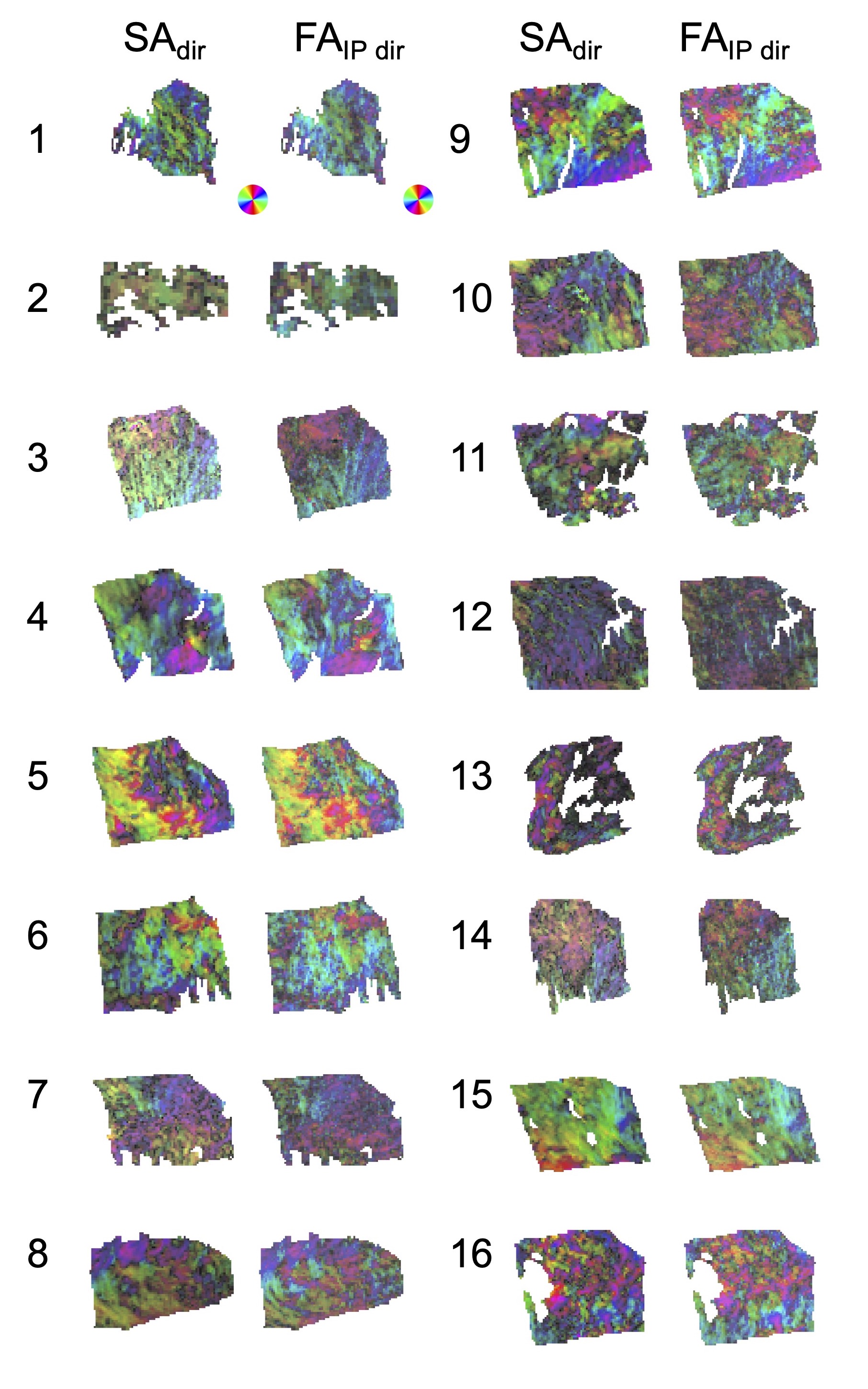


**Supplementary Figure 10. Comparison between directionality of SA and FA_IP_.** The directions are color-coded and the intensity of FA_IP_ and SA is suppressed by scaling the values by $\sqrt{FA_{\mathrm{IP}}}$ and $\sqrt{\mathrm{SA}}$.
